# Social support for collaboration and group awareness in life science research teams

**DOI:** 10.1186/s13029-019-0074-4

**Published:** 2019-07-08

**Authors:** Delfina Malandrino, Ilaria Manno, Alberto Negro, Andrea Petta, Luigi Serra, Concita Cantarella, Vittorio Scarano

**Affiliations:** 1 Dipartimento di Informatica, Università degli Studi di Salerno, Via Giovanni Paolo II, Fisciano (SA), Italy; 2 Consiglio per la Ricerca in Agricoltura e l’Analisi dell’Economia Agraria, Pontecagnano (SA), Salerno, Italy

**Keywords:** Social interactions, Working group awareness, Life science teams collaboration

## Abstract

**Background:**

Next-generation sequencing (NGS) technologies have revolutionarily reshaped the landscape of ’-omics’ research areas. They produce a plethora of information requiring specific knowledge in sample preparation, analysis and characterization. Additionally, expertise and competencies are required when using bioinformatics tools and methods for efficient analysis, interpretation, and visualization of data. These skills are rarely covered in a single laboratory. More often the samples are isolated and purified in a first laboratory, sequencing is performed by a private company or a specialized lab, while the produced data are analyzed by a third group of researchers. In this scenario, the support, the communication, and the information sharing among researchers represent the key points to build a common knowledge and to meet the project objectives.

**Results:**

We present ElGalaxy, a system designed and developed to support collaboration and information sharing among researchers. Specifically, we integrated collaborative functionalities within an application usually adopted by Life Science researchers. ElGalaxy, therefore, is the result of the integration of Galaxy, i.e., a Workflow Management System, with Elgg, i.e., a Social Network Engine.

**Conclusions:**

ElGalaxy enables scientists, that work on the same experiment, to collaborate and share information, to discuss about methods, and to evaluate results of the individual steps, as well as of entire activities, performed during their experiments. ElGalaxy also allows a greater team awareness, especially when experiments are carried out with researchers which belong to different and distributed research centers.

**Electronic supplementary material:**

The online version of this article (10.1186/s13029-019-0074-4) contains supplementary material, which is available to authorized users.

## Background

Next-generation sequencing (NGS) technologies have revolutionarily reshaped the landscape of ’-omics’ research areas. With its significantly lower costs and higher throughput, NGS has played increasing roles in genomic, transcriptomic, and epigenome research. Despite such advances, the development of computing infrastructure and data analysis methods for efficiently processing huge datasets is still behind the speed of data production. The plethora of information that emerges from large-scale next-generation sequencing experiments has triggered the development of bioinformatics tools and methods for efficient analysis, interpretation, and visualization of NGS data. The identification of disease genes by expression profiling or cancer genome projects, as an example, need specific knowledge in sample preparation, analysis and characterization. All required skills are rarely covered in a single laboratory. More often the samples are isolated and purified in a first laboratory, sequencing is performed by a private company or a specialized lab, while the obtained data are analyzed by a third group of researchers.

In this scenario, supporting and enabling communication and information sharing among researchers is a key point to build a common knowledge and to reach the project objective. In general terms, supporting collaboration on the workplaces by means of computers systems is a well-known research field called Computer Supported Collaborative Work (CSCW). The CSCW field is a multidisciplinary area where computer science, information management, sociology, work and organizational psychology converge to explore the many different aspects about the role of the computer to support teamwork. The first aim of CSCW solutions is overcoming time and space limitations among people at different time and/or place to achieve a “virtual co-location” by enhancing remote communication through chat, e-mail, file sharing, audio and video conferencing, and so on [1]. Moreover, CSCW aims to improve group awareness by providing a clear understanding of the current state of the project and of the required and expected steps that have to be performed at a later stage [2].

Therefore, the objectives of CSCW meet the need of supporting teamwork in Life Science research groups, where the continuous interaction and structured communication integrated with data analysis tools and storage is the real added value for the project goals. However, the introduction of collaborative tools in work practices is not painless. Often, domain expertise are needed to effectively introduce computer supported collaboration in specific areas, in order to design functionalities useful and suitable for the domain specific context. Of course, the Life Science research field requires specific efforts to support teamwork. Additionally, the collaborative tools should not involve additional work for users. A poor adoption of these tools, in fact, could become a real risk, if users warn the perception of being overworked.

Given this scenario, our aim is to support collaboration and group awareness in Life Science research teams by integrating a well-known application (Galaxy), usually adopted by researchers, with a vocational social environment where users can share information and achieve an overview on the performed activities. The integration with a well-known application increases the chance of its adoption, by limiting the efforts required for the end users. In the following, we first present our initial analysis about the existing communication and collaboration practices in biological research laboratories. This analysis confirms the need of a greater support for collaboration and information sharing practices. Then, we present ElGalaxy, the system we developed to support collaboration and information sharing among researchers.

## CSCW in life science research teams

In order to evaluate the communication and collaboration practices within the researchers teams, we have conducted an online survey, prepared by using Google Forms^1^. The questionnaire (Additional file 1) is available online^2^. We advertised the survey on SEQanswers^3^, an international community focused on next generation genomics, and on BITS^4^, an Italian assocation of bioinformatics studies. Moreover, we contacted about 50 people by email asking them to answer to the survey. We also asked them to contact other potential interested people. As a result, we collected answers by 32 research centers working in the Life Sciences field.

The questionnaire was composed of three sections aiming firstly to identify the phases of a biological experiment, then, to define the composition of the teams in each phase, and finally, to understand the information sharing mechanisms among the teams. In the following we briefly describe the sample that took part at the study and then we present the results collected for each section.

### Participants demographics

**Fig. 1:**
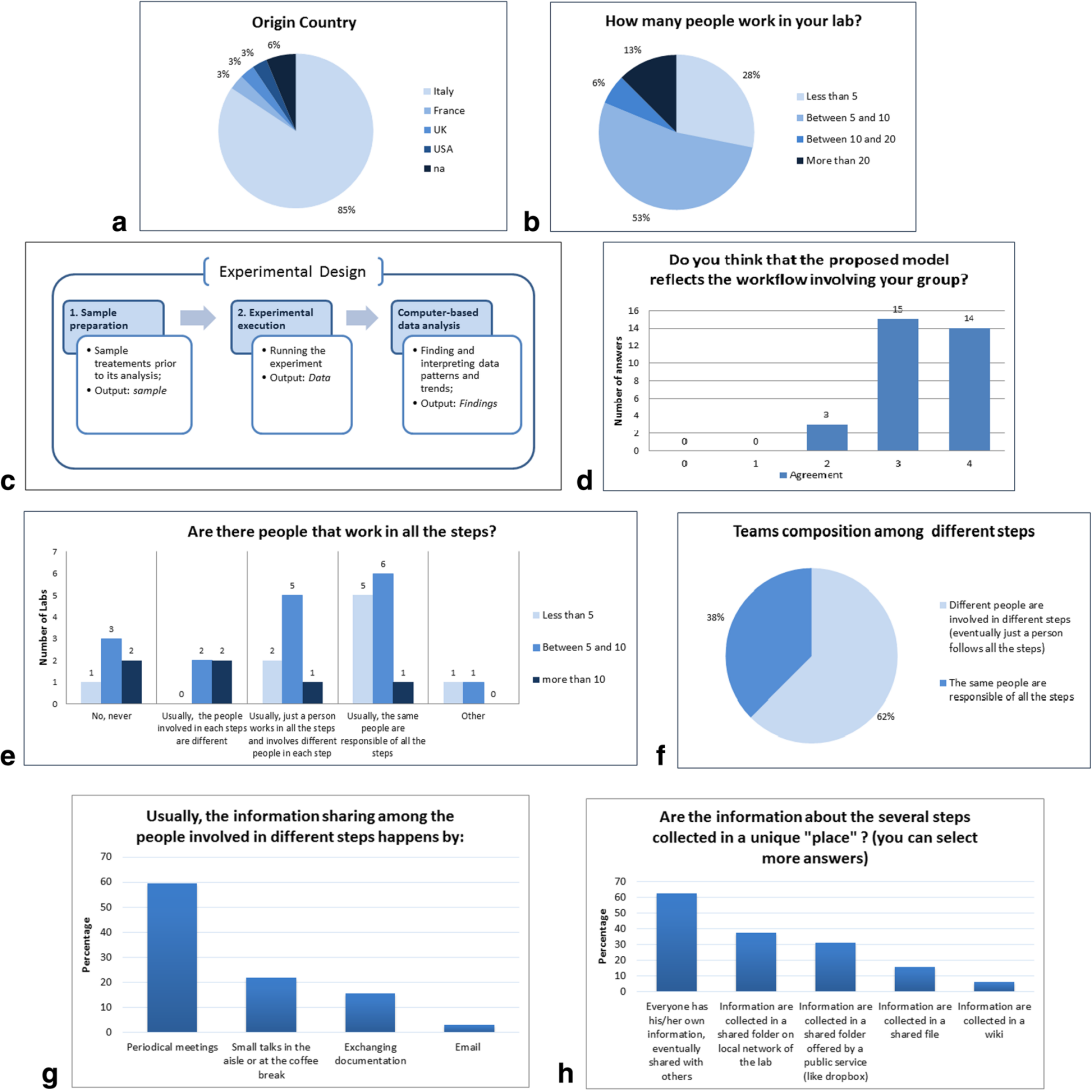
Online Survey results. **a** Users’ origin countries breakdown. **b** Size of teams: a wide majority has less than 10 people. **c** The Model of work phases for biological experiments that we defined and proposed to users. **d** Users’ agreement about the proposed model of work phases. **e** Team composition: small labs have the same people in all the steps, larger labs have different people in different steps. **f** The majority of labs has different people involved in different steps, with eventually one person in charge of supervise all the steps. **g** Information sharing happens mostly in periodical meetings and small talks in the aisle. **h** Information sharing tools: the majority of people collects his/her information, and eventually share it in common folders (public or private)

The questionnaire has been answered by 32 users from different laboratories. As shown in Fig. 1a, the most part of participants comes from Italy (85%) while the remaining from France, UK, and USA. 6% did not specify the country, and generically provided the Europe option, with regard to the question about the origin geographical area. With regard to the size of researchers teams, as shown in Fig. 1b, more than a quarter of the labs (28%) has less than 5 people working there; more than half of the labs (53%) has between 5 and 10 people working there, while 19% has more than 10 people working there (6% has 10-20 people, 13% has more than 20 people).

### Experiment phases

In order to introduce collaborative tools in an effective way, our first step was to define a model with a block schema of project activities (Fig. 1c). In our model the first phase includes the sample collection, extraction, purification or enrichment; the second block consists of the data acquisition, by massively parallel sequencing or, as an example, high-throughput screening; the final phase involves data analysis and features detection, through the execution of several distinct computer programs.

Users expressed their agreement about the model that we proposed. The question required a rating on a 5-point scale, with 0 and 4 as anchors ends. The sequence of phases that we designed has been largely approved (Fig. 1d): almost all the users have agreed with the proposed schema (91% agreed; M=3.3; SD=0.4).

### Teams composition

The team composition in each phase is relevant to understand if different people are involved in different steps: in this case, they need to communicate and exchange information about activities carried out in each phase.

In details, the involvement of people in different phases depends on the size of the lab (Fig. 1e): labs with fewer individuals require their involvement on several phases, while labs with larger teams can assign different phases to different teams (even if this is not always the case).

We also collected two “Other” answers. Specifically, one of the participants stated that: “*It is very beneficial to have at least one people involved in all the steps at least to provide the appropriate feedback*”; therefore, in the overall analysis shown in Fig. 1f, we evaluated this answer as “different people in different steps with just one person following all the steps”. We want also to emphasize that having one person following all the steps is felt as a necessity, not because that person has the skill to lead the experiment, but because that person can collect information about all the activities. Therefore, it is not the better choice in terms of work skills and competencies but it is the better choice in terms of information collection.

The second user which provided the “Other” answer, stated that: “*My group is not involved into biological experiments*”; in this case the lab was in charge just of the work phase 3 (we have elicited this information from the question^5^: *“Usually, which steps are carried out in your laboratory?”*), therefore they collaborate with external centers and have no people involved in the other steps; then in the overall analysis we evaluated this answer as “Usually, the people involved in each steps are different”.

Overall, the question about the team composition in different steps highlights that 38% have the same people involved in all the steps, while a significant majority (62%) have different people in different steps (Fig. 1f). This majority includes who has different people in different steps (always or usually) and who has just one person following all the steps with different people in different steps. This result confirms that supporting communication and information sharing among researchers is becoming a necessity.

### Information sharing

We proposed two questions to evaluate how information sharing normally happens and on which kind of technology support teams can rely on. We found that a large majority shares information in periodical meetings, in small talks in the aisle or at the coffee break (Fig. 1g): more than half of the users (59%) answered that information sharing happens in periodical meetings; the second most frequent sharing information circumstances are small talks in the aisle or at coffee break (22%); both answers (for a total of 81% of answers) rely on the hypothesis that all people involved in the experiment work together in the same lab.

The second question was about the tools used to share information. The results, shown in Fig. 1h, can be summarized as follows: 62% said that everyone has his/her own information, eventually shared with others; 37% said that information are collected in a shared folder on local network of the lab; 31% said that information are collected in a shared folder offered by a public service (like Dropbox); 16% said that information are collected in a shared file and finally 6% said that information are collected in a wiki-like system.

These answers highlight that there is any kind of organization and care of information: for the most part of labs, everyone has his/her information, eventually shared with others through shared (public or private) folders.

Summarizing, the analysis of the online survey confirmed that research activities in Life Science labs are carried out by teams responsible (often) of different tasks; the communication among the researchers happens in periodical formal and informal meetings while information are eventually shared through shared folders. This implies that teams are mostly unaware of the competences and of the activities in the lab. This situation reduces the chances for exchanges, brainstorming, collaboration and the possibility to exploit existing knowledge. This is even more critical if people belong to different organizations and are not co-located.

## Implementation

In this section we first briefly introduce Galaxy and Elgg, the systems upon we built ElGalaxy and then we describe in detail its functionalities. Our idea is to integrate CSCW functionalities with an application usually adopted by researchers: ElGalaxy is the result of the integration of Galaxy (a Workflow Management System) with Elgg (a Social Network Engine).

### Galaxy and Elgg

#### Galaxy

Started in 2005, Galaxy is an open source, Web-based scientific workflow system to build multi-step computational analysis [3–6]. It seeks to increase access to complex computational analyses for all scientists, including those with limited or no programming and administrative knowledge. Large data analyses are possible by using the functionalities provided through the Galaxy’s Web-based graphical user interface (GUI). Using the Galaxy GUI, users can upload their own data or retrieve data from public databases, choose among several analysis tools, set their inputs and parameters and, finally, run tools. Additionally, a workflow editor can be used to create automated, multistep analyses (through the simple drag and drop functionality). Galaxy analyses are completely reproducible. Indeed, all parameters and inputs are permanently recorded, and analyses can be precisely repeated using the GUI. Finally, Galaxy enables users to share and publish their analyses via the Web.

#### Elgg

Elgg is an open source social networking engine that provides a robust framework on which to build customized social environments [7]. For each user, it offers a personal Wall page, with personal posts and related comments of other users. Moreover, it provides the possibility to manage bookmarks, blogging, sharing files, create and sharing pages. Furthermore, Elgg provides a wide set of Plugins, that allow to add extra functionalities. Elgg has a wide community of developers (it hosts a repository of 1000+ open source plugins) and it is used as private social network by (among the others) the NASA, the Australian and British Governments, the Stanford University and the Johns Hopkins University. A full Elgg package is provided under the GNU General Public (GPLv2).

### Functionalities

ElGalaxy has a twofold usage context: it supports individual work in Galaxy and team activities in Elgg. A researcher can use Galaxy as usual and then s/he can share a workflow with his/her team in Elgg. As a consequence, the user’s actions on the workflow in Galaxy (i.e., changes on the workflow, run, saving) trigger notifications to the team members in Elgg. This kind of semi-automated integration reduces the users’ efforts in the adoption of the social environment because they are not in charge of populating the system with contents, which are automatically shared.

The description of ElGalaxy functionalities follows this timeline: *(1)* a user can belong to several groups in Elgg corresponding to work teams; *(2)* the user shares a Galaxy workflow with one of his/her groups and the team members can comment on it; *(3)* each running workflow is shared and can be commented; *(4)* the team is notified about changes on the workflow; *(5)* the team can collect several kinds of shared information (such as files, bookmarks, etc.); *(6)* all the group activities are presented in an activity page.

The user carries out individual actions in Galaxy, while the team actions occur in Elgg. Screenshots about all these activities are available online as supplementary material^6^.

**Fig. 2:**
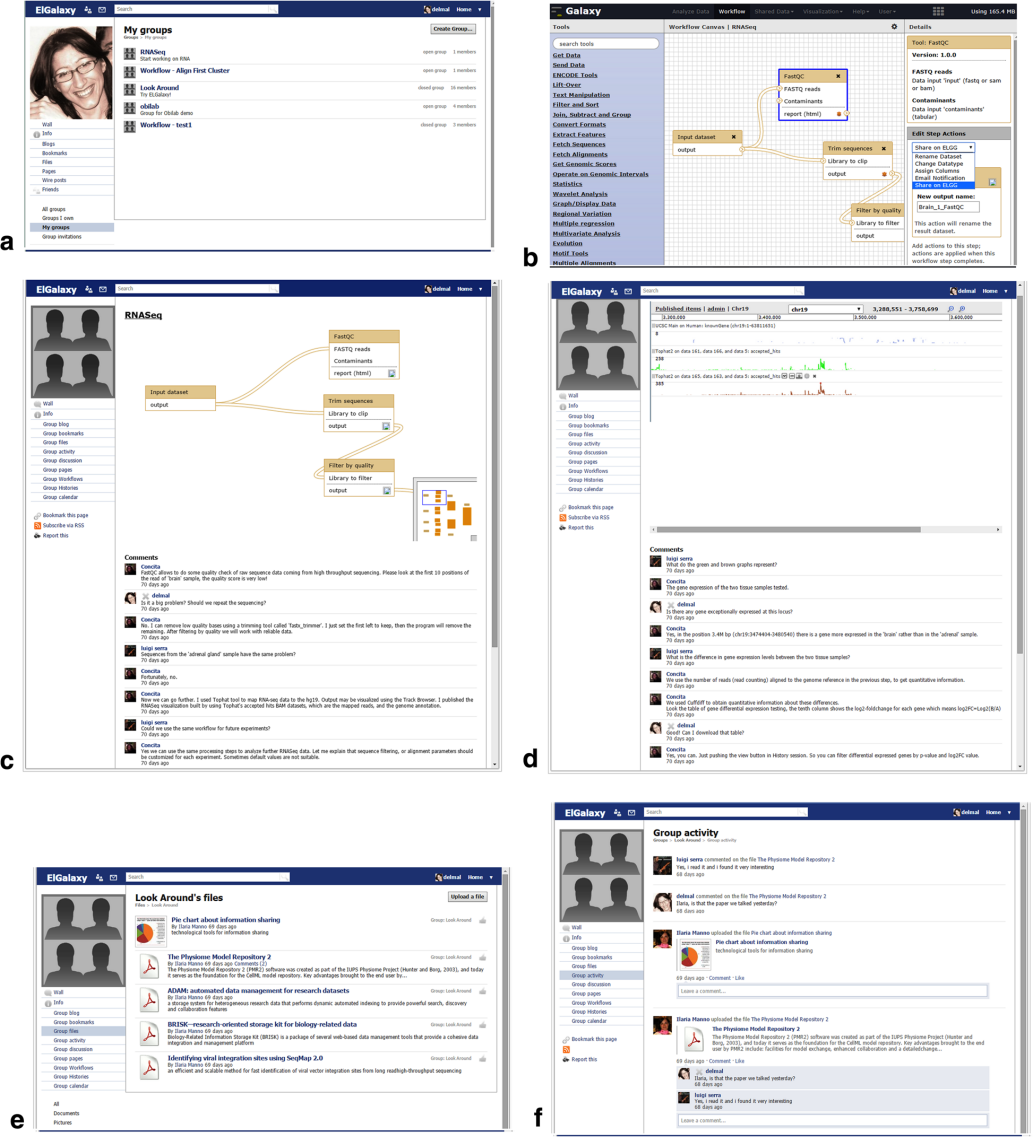
ElGalaxy functionalities. **a** Organization of users in Elgg, each user can be member of more than one group. **b** In Galaxy, a user can share a workflow with one of the groups to which he/she belongs to. **c** In Elgg, each team member can visualize and comment live and active versions of shared workflows. **d** In Elgg, each team member can visualize and comment the histories associated with the execution of shared workflows. **e** Users can share useful documents with other team members. **f** Page of Groups Activities

1. ***User’s groups.*** In Elgg, each user can participate to several groups corresponding to teams involved in specific projects (Fig. 2a). Moreover, the user can create new *groups* and can invite team members.
2. ***Sharing and commenting a workflow***. Each researcher can share his/her Galaxy workflow with a group on Elgg through an explicit action on the workflow (see Fig. 2b). Nothing will be shared without an explicit command of the workflow owner. On the other hand, in Elgg, the team will have a list of all the workflows shared by every team member. Each workflow can be visualized and commented by each team member in order to refine the workflow and to build a common knowledge about the team practices (Fig. 2c). We would to emphasize that the workflow shown in Elgg is not a static image, but the active and live version of the workflow, retrieved in real time from Galaxy.
3. ***Running the workflow and sharing results***. In Galaxy, when a user run a workflow, the system creates a history containing the execution results. If the workflow has been previously shared with a group in Elgg, all the histories coming from its executions will be automatically shared in the same group. Then, in Elgg, team members can see the list of all the histories associated with all the shared workflows. Moreover, they can visualize and comment each history and can see a preview of each step result (Fig. 2d). Sharing histories aims to support team awareness about progress in the work and allows users to discuss about expected or unexpected results.
4. ***Changing the workflow***. In Galaxy, if a shared workflow is modified by its owner, all the team members can immediately visualize the changes in the Elgg environment, which is automatically and permanently synchronized with the analysis activities. It is worth to note that the workflow is just shown in Elgg, therefore no change on the workflow can be made without the intervention of the workflow’s owner in Galaxy.
5. **Sharing docs, information, bookmarks.** In addition to the activities strictly related to the Galaxy workflows, Elgg offers several further functionalities to support collaboration and information sharing within the team. Indeed, it provides a group Blog, where each team member can create pages; a Files page to collect documents useful for the team (Fig. 2e); a Bookmark page to manage a collection of group bookmarks.
6. ***Activities overview***. Besides the functionalities to support communication and information sharing, ElGalaxy aims to improve team awareness, by allowing every one to be updated about the state of the project at a glance. In particular, Elgg provides “Groups” with an “Activities” page where users can receive notifications about workflows changes and executions, uploaded files, pages creation in the blog section and so on; these notifications can be commented by team members (Fig. 2f). All the workflows notifications (changes, run, results) are also added on the calendar of the social environment, so that the group has an overview of activities over the time. These functionalities allow each team member to stay abreast of the group activities easily, even if he/she works in a remote location. Moreover, each user receives notifications on his/her personal “Wall” page about activities of all the groups to which he/she belongs to, so that he/she can be updated about activities of each project.

### Architecture

We integrated Galaxy and Elgg by developing a Bridge software component able to create connections between actions in Galaxy and events in Elgg. The design of the integration is an instance of the generic mechanism presented in a earlier work [8].

**Fig. 3:**
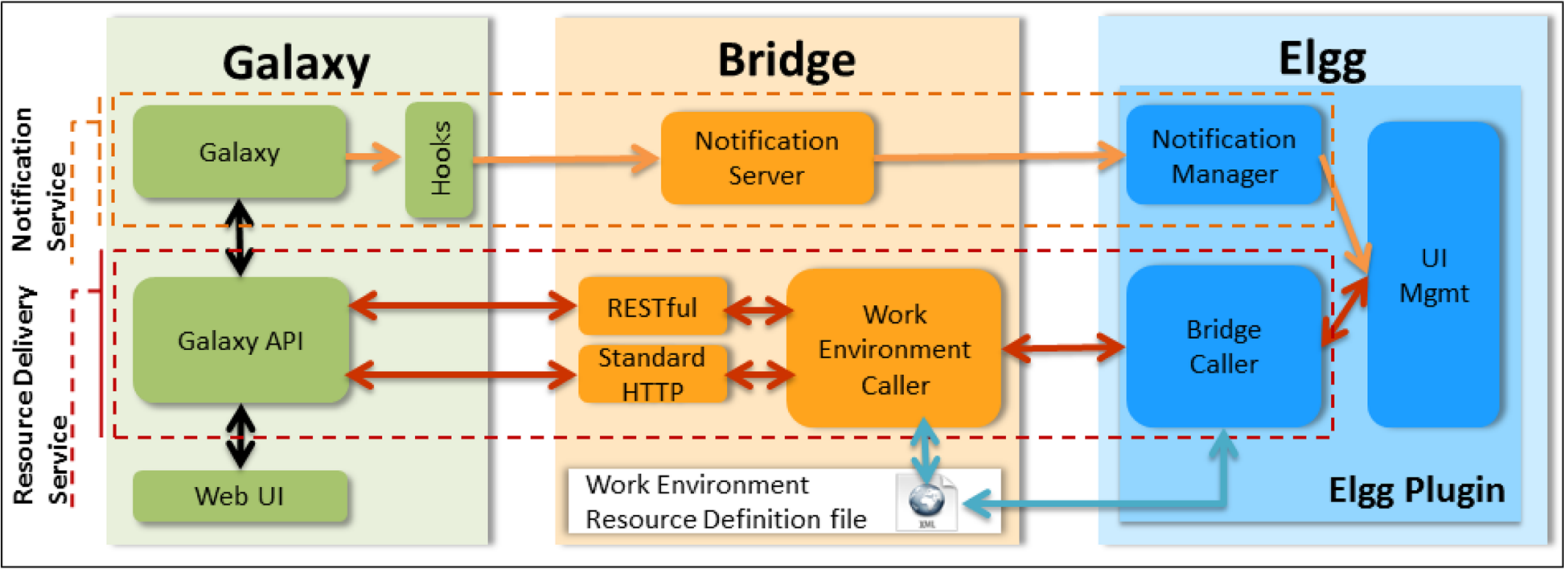
ElGalaxy architecture. Integration of Galaxy and Elgg into ElGalaxy: components and interactions

The communication between Galaxy and Elgg is realized through two different interaction mechanisms (shown in dotted boxes in Fig. 3): the *Resource Delivery Service* and the *Notification Service*. The Resource Delivery Service is responsible of getting from Galaxy the data requested by users through Elgg (for example, the workflow that is shown in the Elgg page). The Notification Service sends notifications from Galaxy to Elgg about some events (i.e., changes and running of workflow, etc.). These services have been implemented by developing different software modules in all the three components of the system, that is, in Galaxy, in Elgg and in the Bridge Component.

With regard to the generic integration mechanism presented in [8], the following modules have been specifically customized: *(a)* the Hooks manager in the Galaxy environment, *(b)* the WERD file on the Bridge Component and *(c)* the whole Elgg plugin.

#### Modules in galaxy

In Galaxy we integrated the Hooks manager module, which is responsible of producing notifications about changes, savings and running of workflows. The Hooks manager intercepts the Galaxy logging activity about changes and savings of workflows, while intercept the running of a workflow step through an action associated with the step execution. Then, the Hooks manager triggers notifications about these events towards the Notification Server (on the Bridge Component).

Finally, Galaxy provides a set of API that allowed us to get data through Web requests (Standard HTTP and RESTful Services). In particular, we used the Galaxy API to get workflows and histories that are shared in Elgg.

#### The bridge component

It includes the modules responsible of transferring resources and notifications from Galaxy towards Elgg. The Notification Server, listens for notifications from the Hooks manager in Galaxy and forwards them to the Elgg Notification Manager. The Work Environment Caller module receives the requests for resources (workflows and histories) from Elgg and gets the corresponding responses through the Galaxy API. Moreover, the Bridge Component contains the WERD file (Work Environment Resource Definition XML file), which contains information to locate the resources available in Galaxy.

#### The Elgg plugin

It is composed of several modules: *(a)* the Bridge Caller, to manage Galaxy resources, *(b)* the Notification Manager, responsible of notifications *(c)* and the UI Management module, responsible of the user interface. The Bridge Caller asks for specific resources (workflows and histories) to the Work Environment Caller in the Bridge Component. Once received the resources, the Bridge Caller makes them available to the UI Management Module. The Notification Manager receives notifications of the user’s actions in Galaxy (changes, save, and running of workflows). Then, it builds the notification and creates entities which can be managed by the UI Management module. The UI Management module is responsible of the integration of data in the user interface of Elgg. It provides synchronous advises and integrates notifications in the “Group Wall” and in the “Group Calendar”; moreover, it leverages the Web UI provided by Galaxy to visualize workflows and the histories to the group.

## Results and discussion

Enabling collaboration and sharing of information among research teams in Life Sciences is a well known necessity. Particular attention has been dedicated to share and organize information in public communities, from several point of view. Among the others, authors in [9] presented a rewarding mechanism to stimulate users participation in knowledge curation and provide also a wide list of Bio-wikis. So far, less attention has been dedicated to support collaboration and communication among researchers working on the same project.

The most notable tool oriented to support collaboration is myExperiment [10, 11]. It is an online research environment that supports the social sharing of bioinformatics workflows. It provides integration with several workflow systems, including the Taverna Workflow Workbench. The collaboration among researchers working on the same project can be supported through the definition of groups and the creation of “Packs” (i.e., collections of items that can be shared as a single entity, such as input data, results, logs, publications). Indeed, the main objective of myExperiment is the creation of a public repository of workflows: the users’ social interaction are focused on discovering and reusing of workflows relevant to their research rather than supporting daily working activities.

To the best of our knowledge, we did not find a system able to support collaboration and communication among researchers in their everyday activities. A research experiment can involve several steps and several people, where each person is responsible of some task and has fully knowledge about its execution. Currently, collaboration and communication within Life Science research teams happen mainly via periodical meetings and informal talks and leverage shared folder to share information, without organization and curation of information.

In this paper we presented our idea of supporting collaboration among people which are working on the same experiment, or set of experiments, and need to share information, to discuss about methods and to evaluate results of the steps carried out. We designed and implemented ElGalaxy with the main objective of supporting social collaboration and team awareness among people working in the same lab as well as belonging to different research centers.

## Conclusion

We presented in this paper ElGalaxy, the result of the integration of a workflow management system with a social network engine. The integration of social interactions in a well established application as Galaxy, allows users to adopt new communication tools without changes in their habits and without additional efforts [12]. Each researcher can use Galaxy as usual and, in addition, he/she can share experiments and data in Elgg to collect comments and hints from his/her colleagues. At the same time, individuals can immediately understand all the activities performed in the laboratory, since ElGalaxy provides an overview of the current state of the project and of the required and expected steps that have to be performed later.

Our vision is that ElGalaxy could become the reference social environment for a team, where each member performs the login into the system as first step in the work day while the logout wraps up the work carried out during the whole day. Its social nature over a small set of contributors represents a live embodied memory of a whole lab knowledge: it is (much) more than a shared repository as it delivers an ongoing representation of team work by storing social interactions and technical discussion in a unique social system. Ongoing works focus on the security aspect. Specifically, we will work in two directions. Firstly we will allow ElGalaxy to work with the https protocol. The second improvement is about a secure authentication through the use of the OAuth 2.0 protocol (https://oauth.net/2/). OAuth 2.0 focuses on client developer simplicity and will provide specific authorization flows for web applications and desktop applications as well as for mobile phones.

Given their positive reaction and the useful suggestions achieved, we are currently planning two exhaustive experimental studies, the first one to evaluate performance [13] and the second one, with a large sample of domain experts, to evaluate the overall system usability and the user satisfaction [14–16].

Additional research directions could explore the integration of other applications besides Galaxy: the generic integration mechanism that we implemented allows to have multiple environments integrated with the social environment. This could enable the social environment to become a dashboard for several kind of team activities.

## Availability and requirements

**Project name**: ElGalaxy

**Project home page**: http://www.isislab.it/projects/ElGalaxy

**Operating system(s)**: Platform independent

**Programming languages**: PHP, Python, Shell scripting

**Other requirements**: PHP, MySQL

**License**: MIT

**Any restrictions to use by non-academics:** None

## Additional file

Additional file 1 The Questionnaire submitted to analyze information sharing mechanisms in Life Sciences teams is available at: http://www.isislab.it/projects/ElGalaxy/Questionnaire.pdf. (PDF 585 kb)

## Data Availability

Project Online Service at: http://www.isislab.it:9380

## Footnotes

1
http://www.google.com/forms/about/

2
http://www.isislab.it/projects/ElGalaxy/Questionnaire.pdf

3
http://seqanswers.com/

4
http://www.bioinformatics.it/

5 Question 9 in the Questionnaire available online

6
www.isislab.it/projects/ElGalaxy/Screenshots.zip
