# Supplementary material for: Social support for collaboration and group awareness in life science research teams
Source: Source Code Biol Med. 2019 Jul 8;14:4. doi: 10.1186/s13029-019-0074-4 (PMC6615102; doi:10.1186/s13029-019-0074-4)
Supplement: Supplementary file 1 — The Questionnaire submitted to analyze information sharing mechanisms in Life Sciences teams is available at: http://www.isislab.it/projects/ElGalaxy/Questionnaire.pdf. (PDF 585 kb) [file 13029_2019_74_MOESM1_ESM.pdf]

Workflow and workgroup in biological research teams

This questionnaire has been prepared by ISISLab, research lab of the Dipartimento di Informatica of the Università degli Studi di Salerno (Italy).

Our aim is to define the workflow and the information sharing mechanisms usually adopted by the research teams in the Biological field. Understanding these aspects is fundamental to develop software systems to support collaboration and information sharing.

We are not specifically interested in your research activity: the questions are generic and concern the work organization only.
All answers will be aggregated in our analysis and will never be published individually.

The questionnaire consists of three sections aiming:
* identify the steps of a biological experiment;
* define the composition of the teams in each step;
* understand the information sharing mechanisms among the teams.

The questionnaire will take approx. 6 minutes.

Your answers will be collected and analyzed for research purpose by
ISISLab - Dip. Informatica, Università degli Studi di Salerno (Italy)

Thank you for your collaboration!

Vittorio Scarano vitsca@dia.unisa.it
Delfina Malandrino delmal@dia.unisa.it
Ilaria Manno manno@dia.unisa.it

http://isis.dia.unisa.it

* Required

Defining the workflow

Here, we aim to define the steps of a Biological experiment.

1. **Do you think that the following model reflects the workflow involving your group?** *

Mark only one oval.

	0	1	2	3	4	
not at all	⬭	⬭	⬭	⬭	⬭	completely

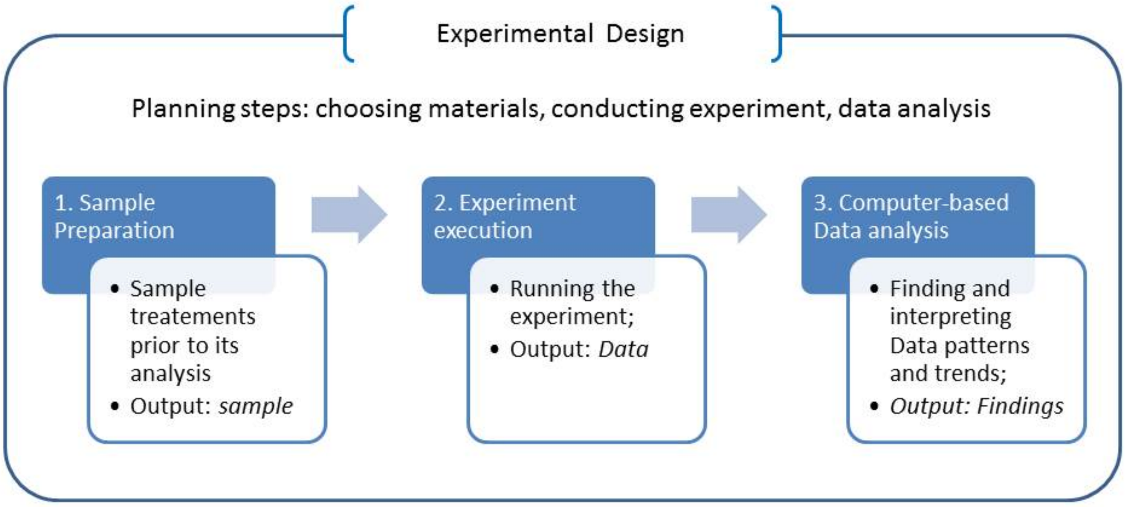

2. **Can you indicate significative differences between your workflow and this model?**

....

....

....

....

....

Work teams

Here we aim to define the organization of the teams involved in each step.

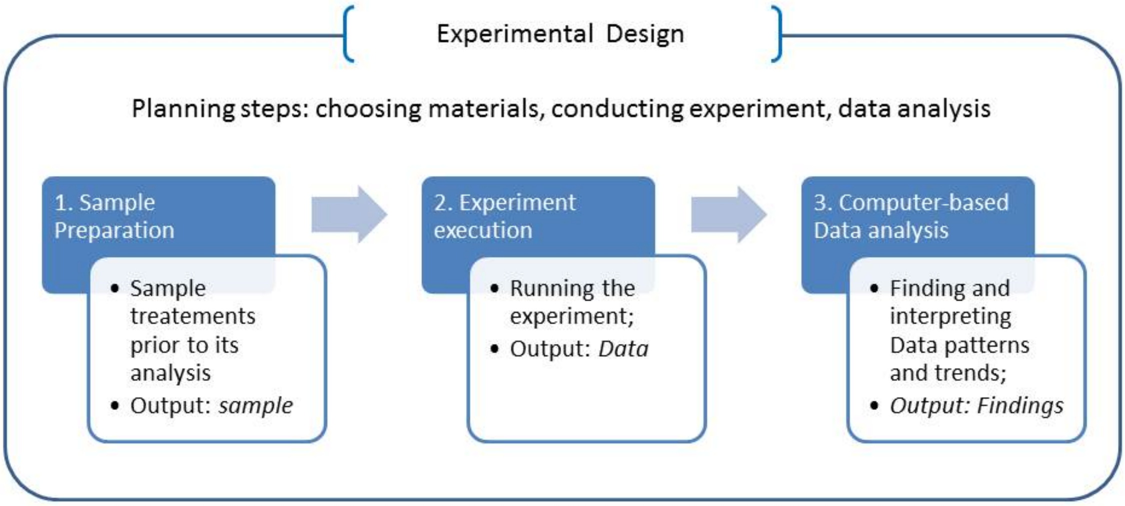

3. **How many people are there in your lab?** *
   Mark only one oval.

   - less than 5
   - between 5 and 10
   - between 10 and 20
   - more than 20

4. **In your lab, are there the skills to execute all the steps?** *
   Mark only one oval.

   - yes
   - no

5. **Do people involved in the step 1 also work in the step 2 ?** *
   Mark only one oval.

	0	1	2	3	4	
never	○	○	○	○	○	always

6. **Do people involved in the step 1 also work in the step 3?** *
Mark only one oval.

	0	1	2	3	4	
never	⬭	⬭	⬭	⬭	⬭	always

7. **Do people involved in the step 2 also work in the step 3?** *
Mark only one oval.

	0	1	2	3	4	
never	⬭	⬭	⬭	⬭	⬭	always

8. **Are there people that work in all the steps?** *
(Please, choose the answer that better reflects your workflow)
Mark only one oval.

⬭ no, never
⬭ usually, the same people are responsible of all the steps
⬭ usually, just a person works in all the steps and involves different people in each step
⬭ usually, the people involved in each steps are different
⬭ Other: ....

9. **Usually, which steps are carried out in your laboratory? (You can select more answers)** *
Check all that apply.

☐ usually, our lab is in charge of step 1
☐ usually, our lab is in charge of step 2
☐ usually, our lab is in charge of step 3
☐ usually, our lab is in charge of all the steps
☐ Other: ....

Information Sharing

Here, we aim to identify the information sharing methods among the teams involved in each step.

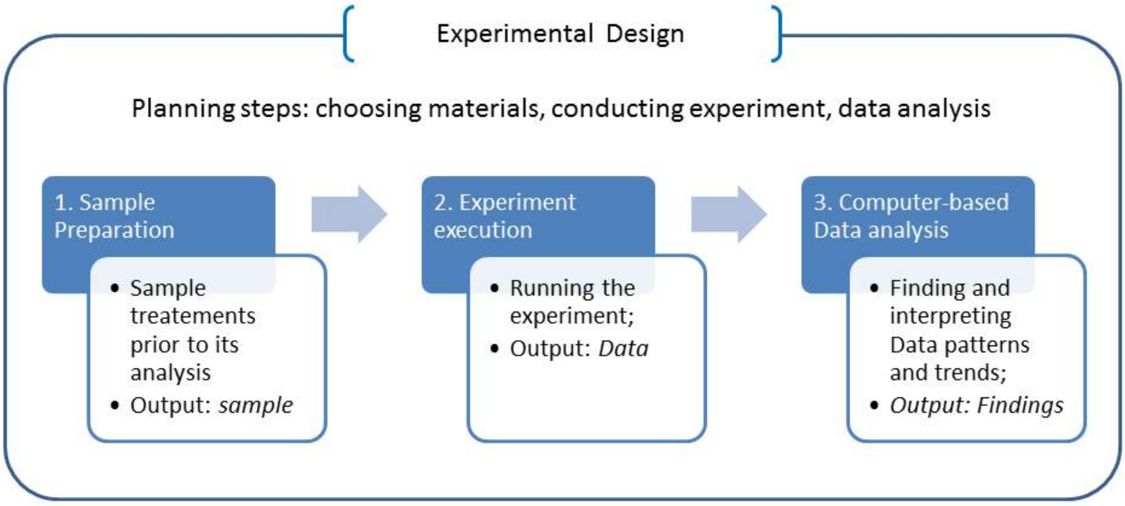

10. **Do you think that the whole experimental design should be shared with the people involved in all the steps?** *
Mark only one oval.

	0	1	2	3	4	
never	⬭	⬭	⬭	⬭	⬭	always

11. **Usually, are people involved in the step 1 informed about analysis and result of next steps?** *
Mark only one oval.

	0	1	2	3	4	
never	⬭	⬭	⬭	⬭	⬭	always

12. **(About the previous question) do you think it is/ would be useful?** *
Mark only one oval.

	0	1	2	3	4	
useless	⬭	⬭	⬭	⬭	⬭	necessary

13. **Usually, are people involved in the step 2 informed about analysis and results of the step 3?** *
Mark only one oval.

	0	1	2	3	4	
never	⬭	⬭	⬭	⬭	⬭	always

14. **(About the previous question) do you think it is/ would be useful?** *
Mark only one oval.

	0	1	2	3	4	
useless	⬭	⬭	⬭	⬭	⬭	necessary

15. **Usually, are people involved in the step 3 informed about the methods adopted in the previous steps?** *
Mark only one oval.

	0	1	2	3	4	
never	⬭	⬭	⬭	⬭	⬭	always

16. **(About the previous question) do you think it is/ would be useful?** *
Mark only one oval.

	0	1	2	3	4	
useless	⬭	⬭	⬭	⬭	⬭	necessary

17. **Usually, the information sharing among the people involved in different steps happens by:** *
(choose the most frequent communication way)
Mark only one oval.

- ⬭ periodical meetings
- ⬭ exchanging documentation
- ⬭ small talks in the aisle or at the coffee break
- ⬭ e-mail
- ⬭ Other: ....

18. **Are the information about the several steps collected in a unique "place" ? (you can select more answers)** *

Check all that apply.

- [ ] information are collected in a shared folder offered by a public service (like dropbox)
- [ ] information are collected in a shared folder on local network of the lab
- [ ] information are collected in a shared file
- [ ] information are collected in a wiki
- [ ] everyone has his/her own information, eventually shared with others
- [ ] Other: ....

About you

19. **Username:** *

(You can use a generic and anonymous username, if you prefer)

....

20. **Geografic Area** *

Mark only one oval.

- ( ) Africa
- ( ) Asia
- ( ) Central America
- ( ) North America
- ( ) South America
- ( ) Europe
- ( ) Middle East
- ( ) Oceania

21. **Country**

....

22. **Email:**

This is optional. You can fill it to receive aggregate results about this questionnaire and if you are available for a possibile further interview.

....

Powered by
Google Forms
